# Data supporting the spectrophotometric method for the estimation of catalase activity

**DOI:** 10.1016/j.dib.2015.12.012

**Published:** 2015-12-17

**Authors:** Mahmoud Hussein Hadwan, Hussein Najm Abed

**Affiliations:** a Chemistry Department, College of Science, University of Babylon, Babylon Governorate, P.O. 51002, Hilla City, Iraq; b Chemistry Department, College of Science, Al-Qadisiyah University, Iraq

**Keywords:** Catalase activity, Hydrogen peroxide, Serum, Ammonium molybdate

## Abstract

Here we provide raw and processed data and methods for the estimation of catalase activities. The method for presenting a simple and accurate colorimetric assay for catalase activities is described. This method is based on the reaction of undecomposed hydrogen peroxide with ammonium molybdate to produce a yellowish color, which has a maximum absorbance at 374 nm. The method is characterized by adding a correction factor to exclude the interference that arises from the presence of amino acids and proteins in serum. The assay acts to keep out the interferences that arose from measurement of absorbance at unsuitable wavelengths.

Specifications table

**Table:** 

Subject area	Biochemistry
More specific subject area	Enzymology
Type of data	Tables, text file, figure
How the data was acquired	Spectrophotometry*,* Shimadzu 1800 spectrophotometer was used in the study
Data format	Analyzed output data
Experimental factors	Serum of one volunteer used without any treatment
Experimental features	Catalase activity was assessed by incubating the enzyme sample in 1.0 ml substrate (65 µmol/ml hydrogen peroxide in 60 mmol/l sodium–potassium phosphate buffer, pH 7.4) at 37 °C for three minutes. The reaction was stopped with ammonium molybdate.
Data source location	Hilla city, Babylon governorate, Iraq
Data accessibility	Data is with this paper

*Value of the data*

• The data presented method that characterizes by adding a correction factor to exclude the interference that arises from the presence of amino acids and proteins in serum.
• The data presented assay that acts to keep out the interferences that arose from measurement of absorbance at unsuitable wavelength.

## 1 Description of the actual data

The following data includes tables, text file and figure that help to measure catalase enzyme activity.

## 2 Experimental design

### 2.1 Principle

Catalase catalyzes the following reaction:

$${\mathrm{2H}}_{2}O_{2}\overset{\mathrm{Catalase}}{\to }{\mathrm{2H}}_{2}O+O_{2}$$

Catalase activity was assessed by incubating the enzyme sample in 1.0 ml substrate (65 µmol/ml hydrogen peroxide in 60 mmol/l sodium–potassium phosphate buffer, pH 7.4) at 37 °C for three minutes. The reaction was stopped with ammonium molybdate. Absorbance of the yellow complex of molybdate and hydrogen peroxide is measured at 374 nm against the blank.

*Reagents*

1. Sodium, potassium phosphate buffer (50 mM, pH 7.4): this buffer is prepared by dissolving 1.1 g of Na_2_HPO_4_ and 0.27 g of KH_2_PO_4_ in 100 ml distilled water.
2. H_2_O_2_ (20 mM) in 50 mmol/l sodium, potassium phosphate buffer: this solution is freshly diluted and standardized daily using a molar extinction coefficient of 43.6 M^−1^ cm^−1^ at 240 nm.
3. Ammonium molybdate (32.4 mmol/l).

*Instrument*:

Shimadzu 1800 spectrophotometer was used in the study.

*Procedure***:** shown in Table 1.

## 3 Calculation

The rate constant of a first-order reaction (*k*) equation is used to determine catalase activity:

(1) $$\mathrm{Catalase}\;\mathrm{Activity}\;\mathrm{of}\;\mathrm{test}\;\mathrm{kU}=\frac{2.303}{t}*[\;\log\;\frac{S{}^{\circ}}{\text{S}-M}]*\frac{\mathrm{Vt}}{\mathrm{Vs}}$$

*t*: time.

*S*°: absorbance of standard tube.

*S*: absorbance of test tube.

*M*: absorbance of control test (correction factor).

Vt: total volume of reagents in test tube.

Vs: volume of serum.

* The present assay uses a correction factor (control-test) to exclude the interference that arises from the presence of amino acids and proteins in the sample that contains catalase enzyme. The absorbance of test tube in procedure is related to two types of compounds, un-reacted hydrogen peroxide and interferences found in serum. The absorbance of control-test tube in procedure relates to interference compounds found in serum only. By subtracting the absorbance of control-test tube from the absorbance of test tube, we eliminate the interference of any compound that may be reacting with ammonium molybdate such as amino acids or proteins. That means the remaining absorbance belong to un-reacted hydrogen peroxide only.

## 4 Data

The method is modified from that elucidated previously by Goth [1] and Korolyuk et al. [2] in which the consumption of hydrogen peroxide is measured spectrophotometrically by a complex reaction with ammonium molybdate at 405 nm or 410 nm. The present method has properties that distinguish them from other assays. The first characteristic includes measurement of absorbance at a wavelength equal to *λ*_max_ (374 nm) which produces results with high accuracy and precision. In an earlier study, Goth [1] measured the absorbance at 405 nm. Goth attributed the reason for this choice to the accessibility of spectrophotometers and filter photometers. Possibly, that choice was good two decades ago. Presently, with the huge progress in spectrophotometric techniques, chemical analysts cannot agree with this explanation [3].

The choice of wavelengths other than 374 nm (such as 405 nm) produces significant disadvantages. It produces unreliable results because of the interference of measurements with each other. It is rare to find a spectroscopic method that uses a wavelength other than *λ*_max_ for chemical analysis. The choice of *λ*_max_ is necessary for various causes. This wavelength distinguishes each compound and gives a description of the electronic structure of the produced complex. It is also used to achieve the highest sensitivity and to decrease deviations from Beer׳s Law [4].

**Fig. 1:**
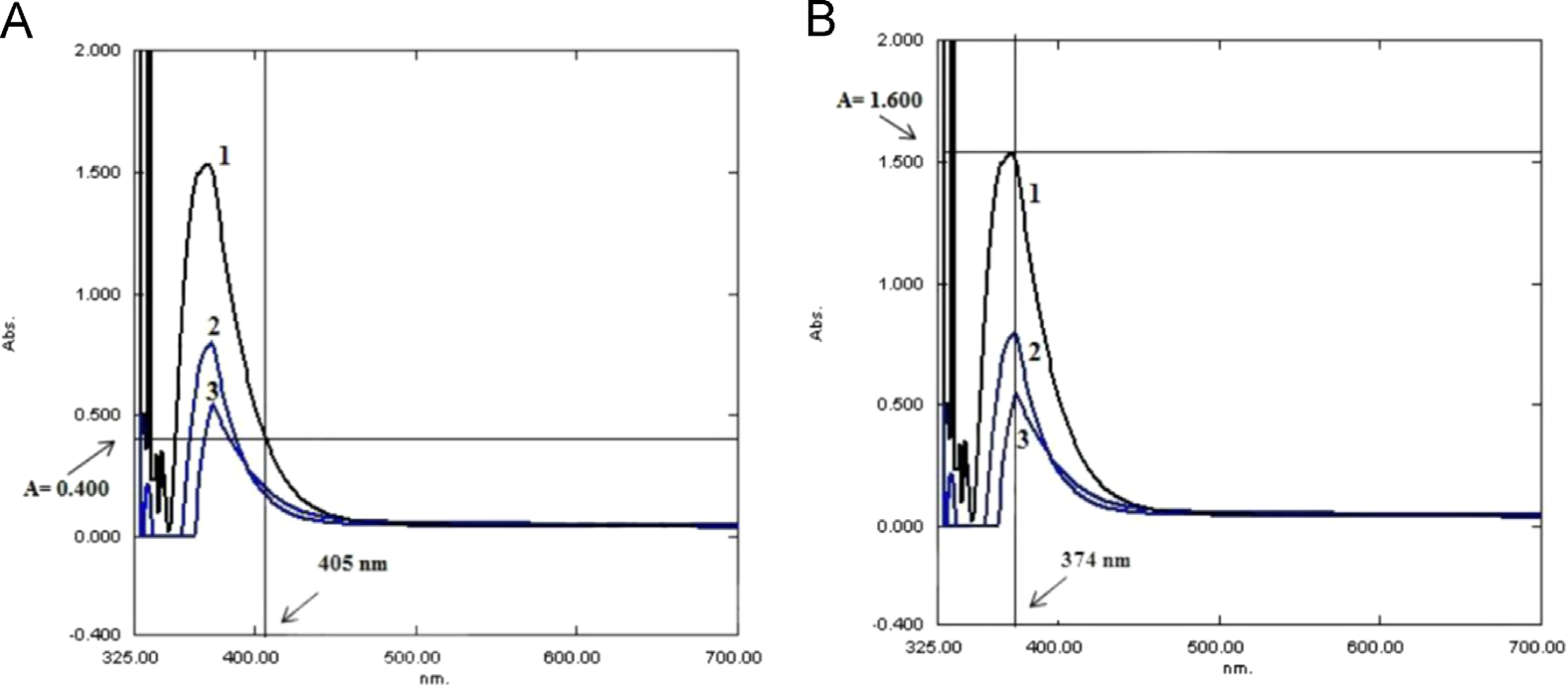
Spectrum of different concentrations of hydrogen peroxide (20 mM, 10 mM and 5 mM, respectively) prepared in 60 mM phosphate buffer after its reaction with 32.4 mM ammonium molybdate.

*λ*_max_ will provide the largest possible accuracy of the measurements because a small change in concentration can provide a greater change in absorbance than other wavelengths. This means that the quantitative analyses are more accurate. Fig. 1 elucidates the difference in accuracy when the absorbance was measured in the *λ*_max_ compared with when it is measured at other wavelengths. Fig. 1(A) represents the wavelength that is used in Goth method, which shows the inappropriate interference between closely spaced levels of the enzyme at wavelength 405 nm, which causes the inability of the Goth method to differentiate between them. In the spectra of Fig. 1(B), we note that the space between curves 1 (20 mM H_2_O_2_), curves 2 (10 mM H_2_O_2_) and curves 3 (5 mM H_2_O_2_) is at a maximum at 374 nm, and at this wavelength the change in absorbance is highest for a given change in concentration. This means that the measurement of concentration as a function of the absorbance is most sensitive at *λ*_max_ wavelength. For these reasons, analysts usually select the wavelength of maximum absorbance for a given solution and use it in the absorbance measurements.

**Scheme 1:**
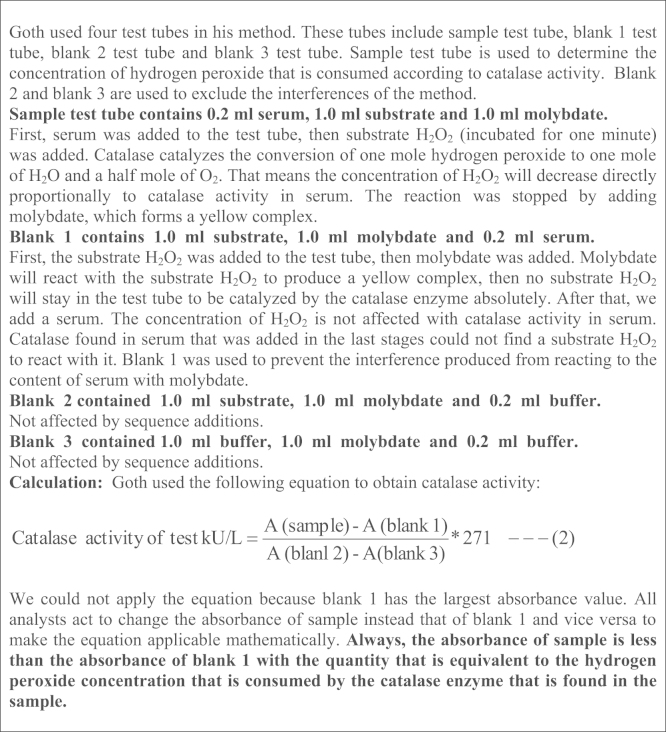
Clarifying the negative aspects in the Goth method.

The second characteristic of the present method includes using the rate constant (*k*) of a first-order reaction equation with correction factor to determine catalase activity. The rate constant of a first-order reaction (*k*) is used to determine catalase activity due to the abnormal kinetics of catalase enzyme. Goth used a special equation to calculate catalase activity and did not use the rate constant of a first-order reaction equation. The equation that was used in the Goth method is a very confusing one as shown in Scheme 1:

The correction factor (control-test) was used in the present method to exclude the interference that arises from the presence of amino acids and proteins in serum. To study the effect of interferences that might disturb the catalase assay, seven volumetric flasks were used and to each one was added one ml of catalase known activity (500 kU/l) [obtained from Himedia (Product Code: TC037)]. Its activity was adjusted according to Aebi׳s method [5] and nine ml of 55.55 μmol/l of one interference only that dissolved in phosphate buffer (50 mM, pH 7.4). The final activity equals to 50 kU/l of catalase with 50 μmol/l of interference. Catalase enzyme activity was measured by the present method (with and without a correction factor). Table 2 indicates the effects of various interferences on the catalase assay. Catalase enzyme activity was not affected significantly by a considerable amount of each interference compound when measured by the present method. However, interferences affected catalase enzyme activity when used method without a correction factor.

Data obtained for a sample of serum by the present method were compared with those obtained by the method of Aebi [5]. Identical samples, buffers, and substrate concentrations were used in both methods.

The data of the present assay provides a good precision (Table 3) and a good correlation with Aebi׳s method (Table 4).

Accuracy of the entire assay protocol was measured by recovery of hydrogen peroxide added to the reaction solution after the end of enzymatic reaction detailed in the Table 5.

## Figures and Tables

**Table 1:** 

Reagents	Test	Control-test*	Standard	Blank
Serum	100 μl	100 μl	–	–
D.W.	–	1000 μl	100 μl	1100 μl
Hydrogen peroxide	1000 μl	–	1000 μl	–
Mix with vortex and incubate at 37 °C for 3 min, after that, add:				
Ammonium molybdate	4000 μl	4000 μl	4000 μl	4000 μl
After that, the tubes were kept at room temperature. Changes in absorbance were recorded at 374 nm against the reagent blank.				

**Table 2:** shows the effects of various interferences on the catalase assay.

Substance	Concentration of substance	Observed catalase activity			
Method without correction factor	Recovery %	Present method	Recovery %
–	0	49.37	–	49.5	–
Albumin	50	41	82	49.2	98.4
Cysteine	50	45	90	49.6	98.8
Histidine	50	43.52	87.04	48.86	97.72
Lysine	50	41.22	87.4	49.7	99.4
Arginine	50	43	86	48.5	97
Methionine	50	46	92	49.2	98.4

**Table 3:** Precision of the assay procedure.

	No.	Mean (±SD) kU/l	CV%
Within-run	20	98.6±2.77	2.8%
Between-run	20	96.33±5.18	5.37%

**Table 4:** Statistical analysis of the values obtained for catalase by Aebi׳s method and present method.

No. of samples	20
Mean of Aebi׳s method	97.7
Mean of the present method	98.6
Mean of both methods	98.15
Regression coefficient B	0.9837
Regression coefficient A	0.0153
Correlation coefficient	0.9839

**Table 5:** Analytical recovery of hydrogen peroxide that is added to the reaction solution after enzymatic reaction stopped.

Present in assay	Equivalents of hydrogen peroxide	Calculated activity kU/l	Observed activity^a^ kU/l	Recovery %
Enzymatic sample	–	–	100	–
Enzymatic sample+hydrogen peroxide	10	110	89	98
Enzymatic sample+hydrogen peroxide	25	125	121	96.8
Enzymatic sample+hydrogen peroxide	50	150	147	98
Enzymatic sample+hydrogen peroxide	100	200	196	98
Enzymatic sample+hydrogen peroxide	200	300	295	97.5

a Mean of triplicate determinations.
